# Characterization of a very rare case of living ewe-buck hybrid using classical and molecular cytogenetics

**DOI:** 10.1038/srep34781

**Published:** 2016-10-04

**Authors:** Alfredo Pauciullo, Christoph Knorr, Angela Perucatti, Alessandra Iannuzzi, Leopoldo Iannuzzi, Georg Erhardt

**Affiliations:** 1 Department of Agricultural, Forest and Food Sciences, University of Torino, Largo P. Braccini 2, 10095 Grugliasco (TO), Italy; 2 Institute for Animal Breeding and Genetics, Justus-Liebig-University Ludwigstraβe 21b, D-35390 Giessen, Germany; 3 National Research Council (CNR), ISPAAM, Laboratory of Animal Cytogenetics and Gene Mapping, via Argine 1085, 80147 Naples, Italy; 4 Department for Animal Science, Biotechnology and Reproduction of Livestock, Georg-August-University, Burckhardtweg 2, D-37077 Göttingen, Germany

## Abstract

The natural occurrence of live hybrid offsprings between sheep and goats has been documented in literature, however all the studies have reported the mating of goats with rams, whereas the reciprocal cross was never documented. This study reports on a very rare case of interspecies hybridization occurred between a ewe (2n = 54, XX) and a buck (2n = 60, XY). The hybrid, born in a German flock under natural conditions, is characterised by an intermediate karyotype (2n = 57, XX). The CBA-banding has shown 3 metacentric and 54 acrocentric chromosomes, whereas the GTG- and RBA-banding have revealed that the autosomes involved in the hybrid combination were CHI1, 3; CHI2, 8 and CHI5, 11 corresponding to the metacentric chromosomes OAR1, OAR2 and OAR3. A tri-colour FISH using chromosome paintings and BAC probes has validated this arrangement. A further FISH analysis has been carried out to analyse the telomeres, which showed a normal structure. Nucleolus organiser-bearing chromosomes were identified as pairs OAR1p(CHI3), OAR2q(CHI2), OAR3q(CHI5), OAR4(CHI4) and OAR25(CHI28), and nuclear associations were found. Sex chromosomes were correctly arranged. The odd number of the karyotype might be responsible for a reduced fertility as consequence of the incorrect chromosomal pairing and/or segregation during the meiosis.

In mammals, interspecies hybridization occurs rarely under natural conditions. This is mainly due to natural barriers or isolating systems, which prevent mating, fertilization and/or development of viable hybrids from animals of different species^1^. Cytogenetic incompatibility is one of the causes of embryo death due to incorrect chromosome paring during the zygote formation and/or aneuploidy occurrence during the zygote division. In most of the cases, interspecies hybrids are hypo-fertile or sterile due to the genetic imbalances at the chromosomal level (incorrect segregation), and at the molecular level, (altered genetic products due to hypo-or hyper-numbered genes copies). Nevertheless, systematic breeding of interspecific hybrids has been favoured to better take advantage of the desirable characteristics of the parental species. A typical example is represented by the mule (*Equus mulus*, 2n = 63), a sterile hybrid obtained by the mating of a domestic horse (*E. caballus*, 2n = 64) with a donkey stallion (*E. asinus*, 2n = 62), normally used as draft animal for its hardiness.

Domestic sheep (*Ovis aries*, 2n = 54) and domestic goats (*Capra hircus*, 2n = 60) are considerably different in both the number and morphology of their chromosomes. Although they show the same fundamental chromosome number (FN) 2n = 60, they do not readily interbreed. However, information of such interspecies mating exists^2^,^3^,^4^,^5^,^6^,^7^, and also experimentally induced hybrid pregnancies have been described^8^,^9^,^10^,^11^.

The hybrid pregnancies are normally lost by 6–8 weeks of gestation mainly for the effect of placental failure due to a maternal immune reaction and cytogenetic incompatibility during zygote division and/or embryo development^4^,^11^,^12^,^13^. In addition, the direction of the cross is important for the outcome of the pregnancy. In fact, the fertilization of caprine oocytes by ovine sperm is more successful than the reciprocal cross^11^,^14^. Warwick and Berry^15^ have postulated that the crosses between ewes and bucks are invariably sterile. Bowermann and Hancock^16^ have observed a very low incidence of successful fertilizations between ewes and bucks, whereas Kelk *et al*.^11^ have reported a total lack of fertilization in ewe-buck crosses.

A healthy female hybrid ewe-buck was born under natural conditions in a small German flock near to Göttingen (Lower Saxony, Germany) in March 2014. The breeder stated that sheep (Leinetal Schaf) and goats (Harzer Ziege) are separated from each other in his farm. However, during the mating season, sexually active bucks with pronounced intense odour are regularly used to stimulate the sexual behaviour of the sheep.

To the best of our knowledge, the animal is the first and only living and healthy hybrid after natural mating of a ewe with a buck. Here, we fully characterise the animal by classical and molecular cytogenetic analysis. In fact, only conventional karyotypes have before been published for goat-ram hybrids so far^4^,^6^, whereas G- and R- bands have been reported only for a doe x ram hybrid case as described by Cribiu *et al*.^17^, but with a poor banding resolution since it was obtained in contracted chromosome preparations.

## Results

**Figure 1:**
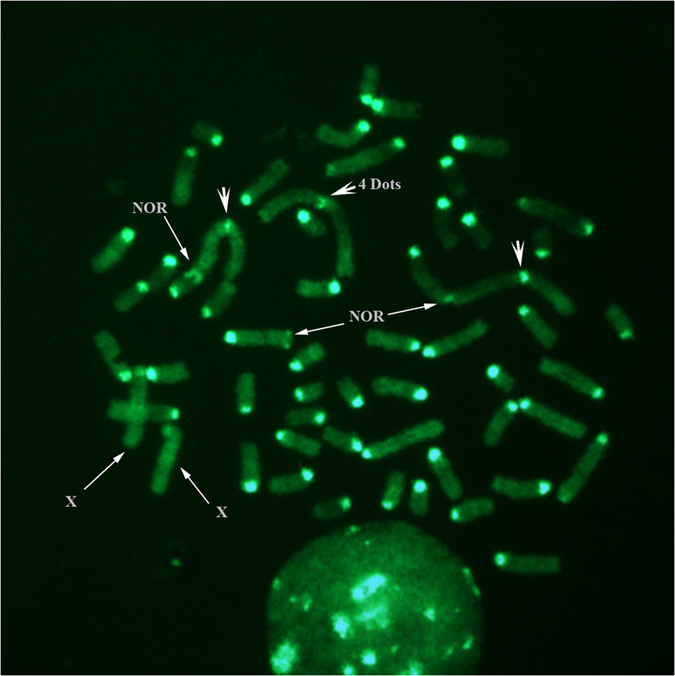
Hybrid’s C-banding by acridine orange staining. CBA-banded metaphase showing prominent constitutive heterochromatin block (C-bands) with the exception of X chromosomes and, in some instances, metacentric autosomes showing C-bands resolved into four small bands/dots (wider white arrows). Occasionally, Nucleolar Organizer Regions (NORs) and association among chromosomes were also visible.

The three investigated animals were karyotyped. The assessment of the conventional chromosome preparations showed karyologically normal parents with a chromosome number of 2n = 54, XX for the ewe and 2n = 60, XY for the buck. Their offspring showed an intermediate karyotype characterised by 2n = 57, XX chromosomes in total (3 metacentric autosomes and 54 acrocentric chromosomes), as confirmed by CBA-banding karyotype (Fig. 1). In this respect, all autosomes showed a distinct and entire centromere (heterochromatin block normally present in sheep and goat) in all observed metaphases. The regions were uniformly intense, whereas in some preparations (in a few chromosomes) this dense region was resolved into two bands and gave the appearance of four dots, as well as occasionally it was possible to distinguish nucleolar regions and the corresponding chromosome association (Fig. 1). Small C-bands were visible for the metacentric chromosomes compared to the other autosomes, whereas both X chromosomes had no distinctive centromeric heterochromatin (Fig. 1).

**Figure 2:**
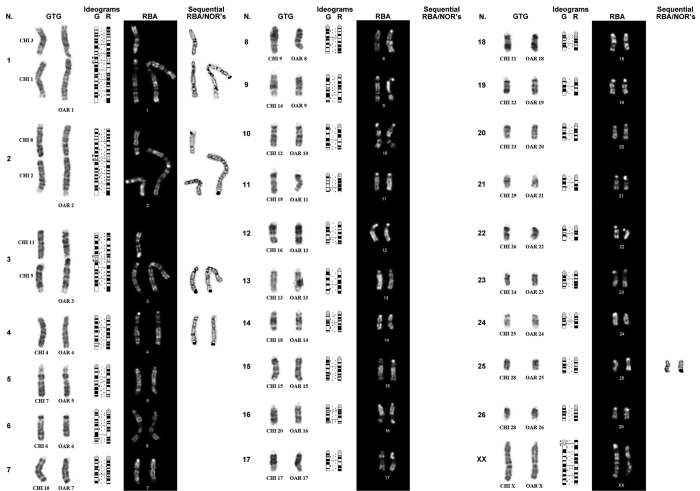
Full karyotype of the hybrid animal. Cytogenetic characterization of the ewe-buck hybrid case. Each individual pair of chromosomes was organised according to sheep karyotype by GTG-banding, ideograms G and R, RBA-banding and sequential RBA/silver staining.

**Figure 3:**
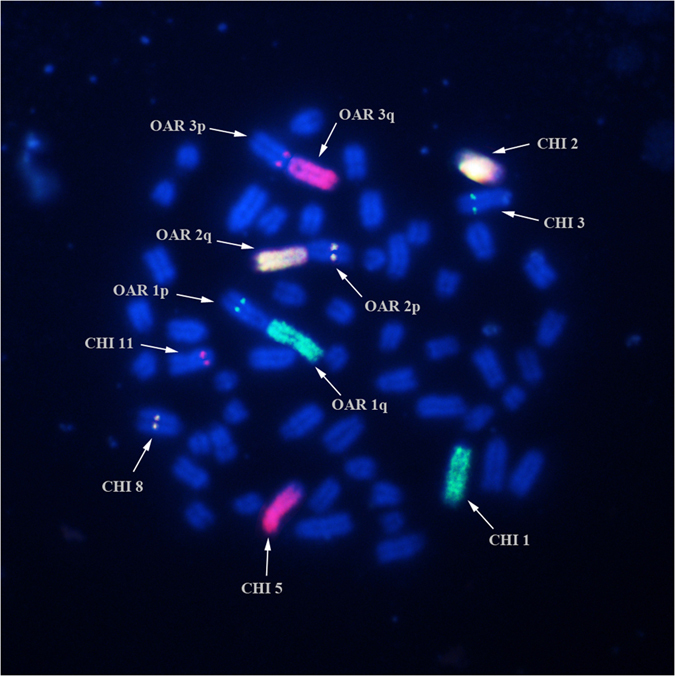
Tri-colour FISH on conventional metaphase spread of the ewe-buck hybrid using specific chromosome paints (spread signals) and BAC probes (dot signals). The painting BBU1q (green) hybridizes on both OAR1q and CHI 1, the painting BBU2q (yellow) identifies both OAR2q and CHI2, whereas the painting BBU4q (red) maps on both OAR3q and CHI5. The BAC 183J23 (marker *LEPR*-green signals) hybridizes on both OAR1p33 and CHI 3q33, the BAC 70B4 (marker *IFN1@* -yellow) identifies OAR 2p15 and CHI 8q15, whereas the BAC 286F8 (marker *LGB* gene-red) maps on both OAR 3p28 and CHI 11q24.

The GTG- and RBA-karyotypes were aligned according to the sheep ISCNDB standard ideograms (Fig. 2). The autosomes involved in the hybrid combination were CHI (*Capra hircus*) 1, 3; CHI 2, 8 and CHI 5, 11 corresponding to the metacentric chromosomes OAR (*Ovis aries*) 1, OAR2 and OAR3 (Fig. 2). The confirmation of the chromosomes involved in the recombinant karyotype was validated by FISH mapping analysis using 3 river buffalo (BBU *Bubalus bubalis*) painting probes (BBU1q, BBU2q, BBU4q) pooled with 3 BAC probes (183J23, 70B4 and 286F8) in a tri-colour experiment. The probe (BBU1q-green signal) for the chromosome CHI1 mapped on both CHI1 and OAR1q, the yellow signal was generated by the probe BBU2q which mapped on both chromosome CHI2 and OAR2q, whereas the probe (BBU4q) for CHI5 gave a red signal on both CHI5 and OAR3q (Fig. 3). The BAC probes identified the remaining chromosomes involved in the recombinant karyotype. In particular, green signals visible as specific dots (*LEPR* marker) were evident on both OAR1p33 and CHI 3q33; yellow signals from the official marker *IFN1@* identified both OAR 2p15 and CHI 8q15, whereas the last marker (*LGB*) labeled in red allowed to identify both OAR 3p28 and CHI 11q24 (Fig. 3).

**Figure 4:**
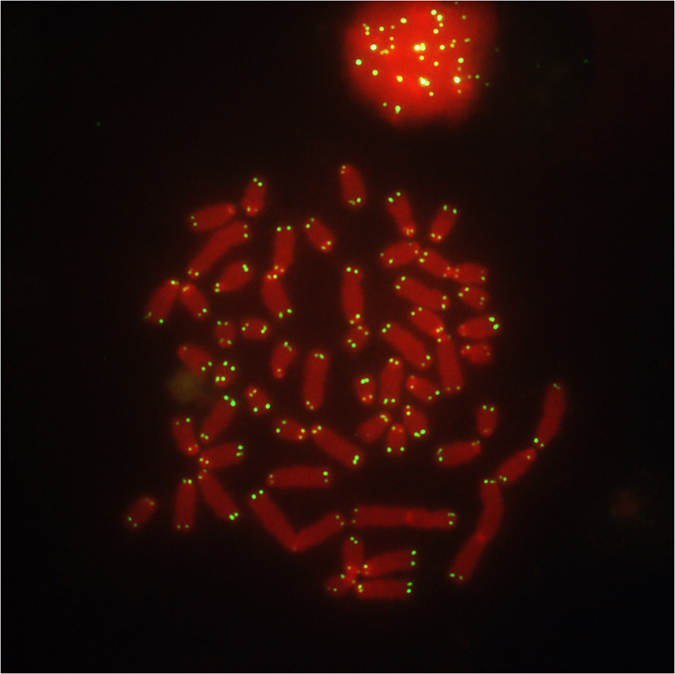
Cytogenetic analysis of the telomeres. FISH with the PNA probe showing telomeric signals in all chromosomes.

**Figure 5:**
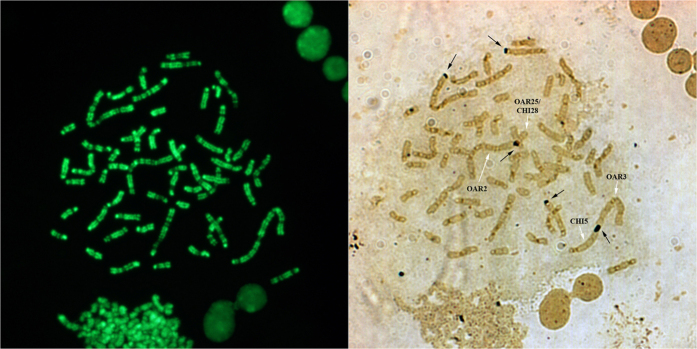
RBA/Ag-NOR banding. Sequential RBA/Ag-NOR metaphase showing active ribosomal regions (NORs) (black arrows) and the nucleolar association between OAR3 and CHI5 and between OAR2 and OAR25/CHI28 (chromosomes indicated by white arrows).

A further FISH analysis was carried out to investigate the telomeres. All chromosomes revealed the presence of fluorescent signals positive for the telomeres (Fig. 4). The investigation of the Nucleolus Organiser Regions (NORs) was accomplished by a classic sequential RBA/NORs banding approach. The last column of Fig. 2 and the Fig. 5 demonstrate the localization of active regions in the telomeric ends of OAR1p (CHI3), OAR2q (CHI2), OAR3q (CHI5), OAR4 (CHI4) and OAR25 (CHI28). Two nuclear associations were clearly visible in almost all analysed metaphases, the first between OAR2 and OAR25/CHI28, the second between OAR3 and CHI5 (Fig. 5).

The sex chromosomes were correctly arranged and no further morphological differences were evidenced by a classical cytogenetic investigation.

## Discussion

Interspecies hybridization of closely related species may generate hybrid offsprings. These events are very rare under normal breeding conditions. Development of the zygotes to term has often not been successful due to cytogenetic incompatibility^15^ and haemolytic disease resulting from maternal antibodies developed against foetal red blood cells^13^. However, the natural occurrence of live hybrid offspring between caprine and ovine species is well documented in literature, although all these studies report on goats mated with rams^2^,^3^,^4^,^6^,^11^,^17^,^18^. The reciprocal cross, in terms of living hybrids, was never reported.

In this study, we describe the occurrence of an alive hybrid offspring born from the cross of a buck with a ewe under natural conditions. The hybrid is a healthy female characterised by a diploid number of chromosomes 2n = 57, XX. All chromosomes paired correctly. The buck acrocentric chromosomes CHI 1 and 3, CHI 2 and 8, CHI 5 and 11 correctly paired to the corresponding ewe metacentric chromosomes OAR1, OAR2, and OAR3, as revealed by resolutive G- and the R-banding patterns as well as FISH analysis.

Ewes bred naturally or inseminated with buck spermatozoa usually fail to conceive, or embryonic development rarely goes beyond the first few stages of cleavage^19^. Bowerman and Hancock^16^ reported one cleaved ovum among 40 collected from 15 ewes bred to bucks, whereas Kelk *et al*.^11^ described the total lack of *in vivo* fertilization of ewes by buck sperm. However, *in vitro* experiments confirmed the ability of the gametes to successfully interact^20^, thus suggesting that the barrier to *in vivo* fertilization involves sperm capacitation. In fact, according to these authors, the direct insemination into the uterus (by-passing the cervix) does not promote fertilization, suggesting that the capacitation of buck spermatozoa in the reproductive tract of the ewe may be disadvantaged^11^. However, the hybrid described in the present study was conceived under natural conditions, therefore (at least limited at this case) no capacitation problems for the buck sperm occurred.

In general, the polarity of the hybridization is described as strongly affecting the success or failure of interspecies crosses also in other species. For instance, Kochhar *et al*.^21^ found that the cleavage rate of buffalo oocytes exposed to cattle sperm was half (40.8%) compared to cow oocytes treated with buffalo sperm (86.3%). Therefore, the origin of the oocyte resulted in a different attitude of the *in vitro* hybrid embryo to develop to advanced blastocyst stages.

It is evident that the ewe-buck hybrid herein described represents a rare combination of positive circumstances like normal sperm capacitation^11^, good polarity of hybridization^21^, good interaction between mitochondrial and genomic DNA^22^, normal activation of zygote genome^23^,^24^, proper placenta formation^24^, absence of haemolytic disease or immune response^24^, and so on. All these events singularly might be responsible for failure in interspecies hybrids development; however in this case, they allowed first the zygote formation, the early stage of embryo development, then the normal growth of the foetus and the adult animal.

**Figure 6:**
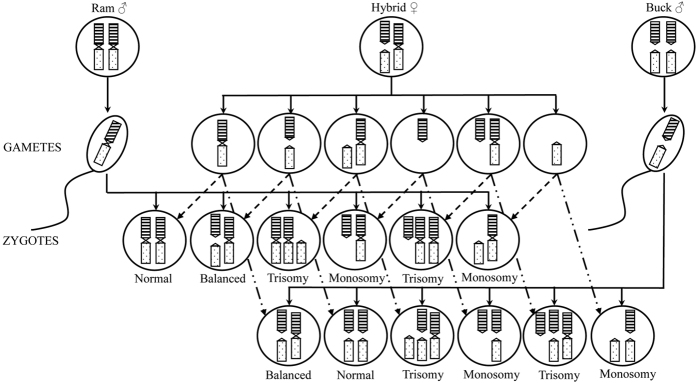
Schematic representation of the oocytes chromosomal segregation for each of metacentric/acrocentric hybrid autosomes and the fertilization with a normal ram or buck spermatozoa. Six different types of zygotes can be produced for each metacentric/acrocentric hybrid chromosomes giving only 1 normal and 1 balanced embryo, whereas the others carrying hyper- or hypo-ploidies.

The analysis of the C-bands showed very well marked centromeric heterochromatin, particularly in the case of the acrocentric autosomes, whereas small blocks (often scarcely detectable in some metaphases to appear almost like 4 distinct dots) characterised the 3 metacentric chromosomes (Fig. 1). The latter characteristic is typical of the ovine C-band pattern, described as evolutionary events resulting from a relatively recent Robertsonian fusions involving two acrocentric chromosomes with loss of centromeric heterochromatin^25^. The imbalance in the amount of centromeric heterochromatin between the 3 metacentric chromosomes and the corresponding acrocentric autosomes in the hybrid animal might be a very probable cause of incorrect pairing and/or segregation during the meiosis division. This event normally occurs in hybrid animals, whose fertility results to be greatly reduced for the production of aneuploid oocytes. In this case, each triplet of chromosomes involved in pairing gives rise to normal gametes in the ratio 1:6 (Fig. 6). Therefore, the probability to have normal gametes and, as a consequence, normal embryos after fertilization with ram or buck normal spermatozoa is equal to [(1/6)^3^]*100 = 0.463% for the three triplets of involved chromosomes.

The reduction of the fertility (or sterility) is well-known for other hybrids (for instance *Bos taurus* x *Bos grunniens* or *Equus caballus* x *Equus asinus* male offsprings), instead no data are currently available on the chromosome segregation and no information is known about the real incidence of gamete aberration for this rare ewe x buck hybrid animal. In this respect, the painting probes and the BAC marker used in this study for chromosome identification would represent the best solution for the analysis of the hybrid oocytes. In fact, the great condensation level of meiotic chromatin does not allow a resolutive banding, thus limiting the karyotyping to a conventional Giemsa staining or C-banding which prevent the chromosome identification^26^,^27^,^28^. On the contrary, as already described in other species, the use of chromosome paints gives unambiguous results in the detection of aneuploidies in oocytes^29^,^30^,^31^.

The hybrid was investigated also for the presence of active NORs. Out of 30 silver stained analysed metaphases, the majority of the cells (more than 50%) showed five NORs (Figs 2 and 5). The identified chromosomes agreed with the findings reported in goat^32^ and sheep^33^ respectively. Conversely, our data only partially agree with the previous identification of NORs in goat reported by Di Meo *et al*.^34^, in particular, we could not find the active NOR on CHI6. However, this discordance is due only to the different chromosome nomenclature^35^, definitively clarified ten years later with the international standard^36^.

In conclusion, the present study is the first report providing specific cytogenetic information on the chromosomal constitution of a very rare case of a ewe-buck hybrid born under natural conditions, including the GTG-, RBA- and C-banding patterns and the identification of five pairs of Ag-NOR bearing chromosomes. The molecular investigation using specific chromosome paints and official marker BAC probes validated the mixed karyotype (2n = 57), with 3 metacentric and 54 acrocentric chromosomes, whereas the use of PNA probe showed a normal arrangement of telomeres.

Considering the odd number of the karyotype, the hybrid animal might show reduced fertility for the effect of the incorrect chromosomal pairing and/or segregation during the meiosis division. However, the real level of aneuploidies in the oocytes of the hybrid animal is unknown and further molecular cytogenetic analysis, as well as information on genome sequencing, transcriptome and mtDNA investigation is necessary to clarify this and other aspects of such a unique hybrid animal.

## Methods

### Ethics approval

The study was done according to the German Animal Welfare Law (released on 05/18/2006, last changes on 07/28/2014). On the basis of this law, no further notification or approval by the Animal Protection Unit of the Regional Council of Göttingen (Germany) was necessary for the study.

Furthermore, procedures were also in accordance with the ethical standards of the Italian national ethics committee on research on animal science of the 7^th^ June 2011. The experimental protocols were approved by the institutional committee on the ethics of animal experiments of National Research Council of Italy (Protocol Number: 00000082–25/01/2016).

### Cell cultures and karyotyping

Whole blood samples were collected from the jugular vein of the ewe-buck hybrid and its parents (dam sheep and sire goat), using sterile vacutainer tubes containing sodium heparin as anticoagulant. About 1 ml of whole blood sample was added to the culture mix composed of 7 ml of RPMI medium, enriched with fetal calf serum (20%), L-glutamine (300 μg/ml), antibiotic-antimycotic mixture (1%) and concanavalin A (20 μg/ml) as mitogen. Culture flasks were incubated at 37.5 °C for 72 h. Cell cultures were treated for conventional (normal cultures) and late-incorporation of BrdU (15 μg/ml) to obtain R-banding preparations. Hoechst 33258 (30 μg/ml) was simultaneously added to BrdU 6 h before harvesting to enhance the R-banding patterns. Both cell cultures were gently agitated once a day and subjected to 1 h of colcemid (0.5 μg/ml) treatment, followed by centrifugation steps, and hypotonic (KCl 75mM) and fixative methanol/glacial acetic acid (3:1) treatments according to Iannuzzi and Di Berardino^37^. Cell suspensions were dropped onto cleaned and wet slides and then air dried.

After the fixation, a part of the obtained metaphases was stained with a 5% Giemsa solution. Another aliquot was treated with 0.05% of trypsin solution and Giemsa staining to obtain the GTG-banding. C-banding (CBA) and sequential R-banding by fluorescence with acridine orange (RBA)/Ag-NOR-staining were performed according to Iannuzzi and Di Berardino^37^.

Banded karyotypes were arranged. Chromosome identification followed the standard ideogram according to the latest international nomenclature for domestic bovides chromosomes^36^.

### Probes production and labeling

Painting probes corresponding to the goat acrocentric CHI 1, 2, 5 and sheep metacentric OAR 1q, 2q and 3q were produced via chromosome microdissection from river buffalo GTG-metaphases by scraping the following homologous chromosomes BBU 1q, BBU 2q, BBU 4q^38^. Microdissected chromosomes were amplified by DOP-PCR following the protocol of Pauciullo *et al*.^39^. Probes were then labeled with Biotin-16-dUTP (BBU 1q and 4q) and DIG-11-dUTP (BBU 2q and 4q) in a second DOP-PCR reaction, using 2 μl of the product used in the first reactions as template. Probes labeled with both modified nucleotides were combined in the same ratio to perform a tri-color FISH.

DNA isolation from the BAC clones 70B4 and 286F8 and the clone 183J23 was carried out according to the alkaline lysis miniprep protocol suggested by the Children’s Hospital Oakland Research Institute (CHORI, Oakland, CA, USA). Two of them (70B4 and 286F8) are official BAC clones^36^,^40^. The first carries the marker *IFN1@* (OAR 2p15-BTA/CHI 8q15), whereas the second carries the *LGB* gene (OAR 3p28-BTA/CHI 11q24). The third BAC (183J23) is unofficial, but it belongs to BAC library CHORI-243 (www.bacpac.chori.org); it maps on OAR 1p33-BTA/CHI 3q33 and it carries *LEPR*^41^.

Approximately 1.5 μg of BAC DNA was combined with 20 μl of 2.5x random primer (BioPrime aCGH Labeling Module, Invitrogen, Germany) in a total volume of 39 μl. Samples were incubated at 95 °C for 5 min and then placed on ice for 5 min. Next, 5 μl of 10x dUTP, 1 μl Exo-Klenow Fragment (BioPrime Module, Life Technology, Carlsbad, CA, USA) and 5 μl (0.6 mM) of Biotin-16-dUTP (for the BAC 183J23) or DIG-11-dUTP (for the BAC 286F8) were added. In order to perform a tri-color FISH, a combination of both modified nucleotides was used for the labelling of the BAC 710B4. All tubes were incubated at 37 °C for 5 h and then used for *in situ* hybridization.

### Fluorescent *in situ* hybridization

Two different FISH analyses were conducted using chromosome paintings combined with BAC probes in a tri-colour hybridization and a PNA probe for telomere analysis.

### Tri-colour FISH

A tri-colour fluorescent *in situ* hybridization was performed according to Pauciullo *et al*.^39^ by using 3 painting probes (BBU 1q, BBU 2q, BBU 4q) and 3 BAC probes (183J23, 70B4 and 286F8) labeled according to the scheme reported in Table 1. All labeled probes were mixed and precipitated in absolute ethanol together with 10 μg of salmon sperm DNA and 10 μg of calf thymus DNA (both from Sigma-Aldrich, Saint Louis, .MO, USA). The pellets were vacuum-dried and then resuspended in 15 μl of hybridization solution (50% formamide in 2x SSC + 10% dextran sulfate) for 1 h at 37 °C. The probes were denatured for 10 min at 75 °C and pre-hybridized for 60 min at 37 °C. Metaphase spreads were denatured for 3 min in a solution of 70% formamide in 2x SSC (pH 7.0) at 75 °C. Denaturation was stopped in a scale 70%, 80% and 96% of cold ethanol and air dried. The hybridization mixture was applied to the slides, covered with 24 × 24 mm coverslips and incubated in a moist chamber at 37 °C over-night. After hybridization, coverslips were removed by a gentle washing step in 2x SSC. The slides were then washed 3 × 4 min in a washing solution (50% formamide in 2x SSC) at 42 °C, followed by 3 additional washing steps for 4 min in 2x SSC at 42 °C and a further step for 5 min in PBST at room temperature. A detection step was carried out for 1 h at 37 °C applying a mixture of fluorescein isothiocyanate-avidin (Vector Laboratories, Burlingame, CA, USA) and rhodamine anti-digoxigenin antibody from sheep (Roche Diagnostics, Milano, Italy) both diluted 1:400 in PBT buffer. Three washing steps were accomplished in 1x PBST for 5 min, each at room temperature by gently shaking. Finally, slides were counterstained with DAPI (4,6-diamidino-2-phenylindole) solution (0.24 μg/ml; Sigma-Aldrich) in Antifade (Vector Laboratories).

### Telomere PNA Probe

A two hours lasting FISH procedure was performed to analyze the telomeres using a fluorescein-conjugated PNA probe mapping on all telomeres (Dako Cytomation, Denmark) according to the manufacturer’s guidelines. Briefly, a pre-treatment in 3.7% paraformaldehyde in TBS was carried out on the slides for 2 min at room temperature, followed by two washing steps in TBS for 5 min each and then cold ethanol scale of 70%, 85%, and 95% for 2 min each. 10 μl of the telomere PNA probe was applied in a marked area, covered with a 24 × 24 mm cover slip and incubated at 80 °C for 4 min on a pre-heated plate. Incubation was carried out in the dark for 30 min at room temperature. Washing steps were accomplished in a pre-heated wash solution at 65 °C for 5 min, followed by the same cold ethanol scale for 2 min each. Slides were finally mounted in Propidium Iodide/Antifade (Vectashield H1300/H1500, Vector Laboratories) and stored in the dark for 30 min before the microscopic observation.

### Microscopic analysis

The slides prepared for banding and for FISH were observed at 100x magnification with a Leica DM5500 fluorescence microscope equipped with DAPI, FITC, Texas Red specific filters, the FITC/Texas Red double filter, and provided with a Cytovision MB 8 image-analysis system (Leica Microsystems, Wetzlar, Germany). Digital images were captured in grey-scale, whereas false colours were created by the image-analyzing system for a reliable evaluation of the painting probes. Approximately 25–30 metaphases were acquired for each slide.

## Additional Information

**How to cite this article**: Pauciullo, A. *et al*. Characterization of a very rare case of living ewe-buck hybrid using classical and molecular cytogenetics. *Sci. Rep.* **6**, 34781; doi: 10.1038/srep34781 (2016).

## Figures and Tables

**Table 1:** Labelling scheme for the achievement of the tri-colour FISH using chromosome painting and BAC probes.

Probe	Painting	BAC	Painting	BAC	Painting	BAC
	BBU1q	183J23	BBU2q	70B4	BBU4q	286F8
Colour	Green		Yellow		Red	
Sheep	1q	1p33	2q	2p15	3q	3p28
Goat	1	3q33	2	8q15	5	11q24
